# Correction: A graphite-modified natural stibnite mineral as a high-performance anode material for sodium-ion storage

**DOI:** 10.1039/d3ra90022c

**Published:** 2023-03-23

**Authors:** Hongliang Li, Mingxiang Deng

**Affiliations:** a College of Chemistry and Chemical Engineering, Central South University Changsha 410083 China; b College of Science, Central South University of Forestry and Technology Changsha 410004 China 511584588@qq.com

## Abstract

Correction for ‘A graphite-modified natural stibnite mineral as a high-performance anode material for sodium-ion storage’ by Hongliang Li *et al.*, *RSC Adv.*, 2019, **9**, 28953–28960, https://doi.org/10.1039/C9RA02663K.

The authors regret errors in the list of co-authors, in the data and corresponding text for Fig. 1b and 5, and in the captions for Fig. 1–5.

Hongshuai Hou and Xiaobo Ji wish to be removed from the authorship of this paper. The experiments, data compilation and writing of this article were carried out by the first author Hongliang Li and the corresponding author Mingxiang Deng, and the article was submitted by Hongliang Li. And they erroneously listed Xiaobo Ji and Hongshuai Hou as co-authors. Xiaobo Ji and Hongshuai Hou only provided some instruments and consumables for the experiments in this paper, so Xiaobo Ji and Hongshuai Hou do not feel their contribution is sufficient to be authors on this paper, and they request to update the authorship of this article to remove Hongshuai Hou and Xiaobo Ji as authors of this paper. The correct authorship is presented herein.

The following sentence should be included in the acknowledgements: ‘The authors thank Hongshuai Hou and Xiaobo Ji for providing experimental equipment and resources.’

The authors regret that the images provided for Fig. 1b and 5 in the original article are incorrect. Corrected versions of Fig. 1b and 5 are provided herein.

**Fig. 1:**
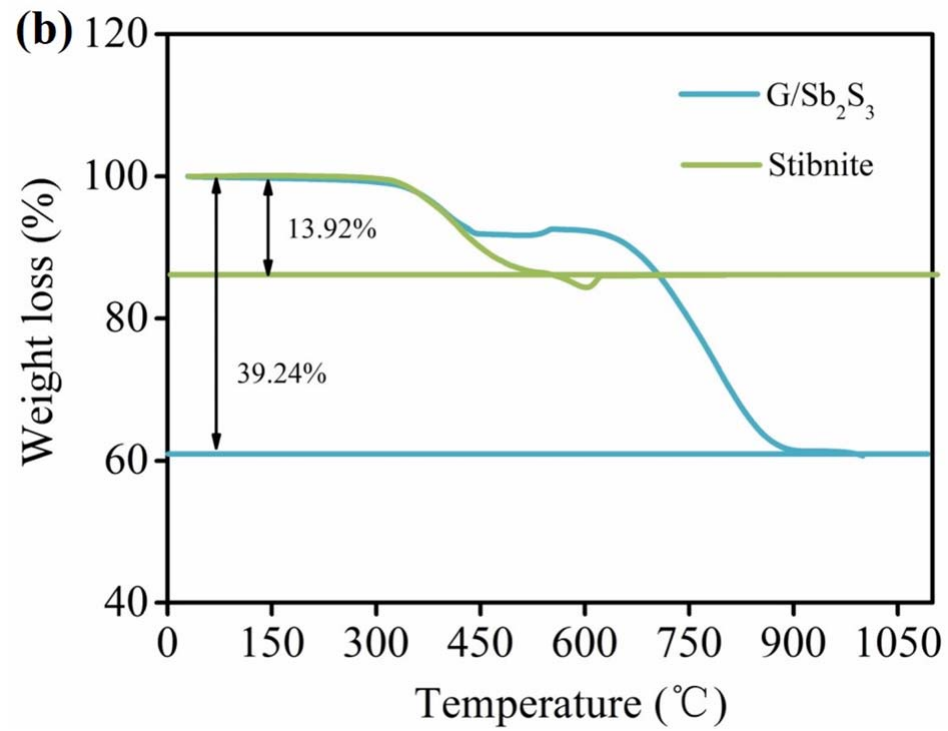
(b) TGA curves of G/Sb_2_S_3_ and stibnite.

**Fig. 2:**
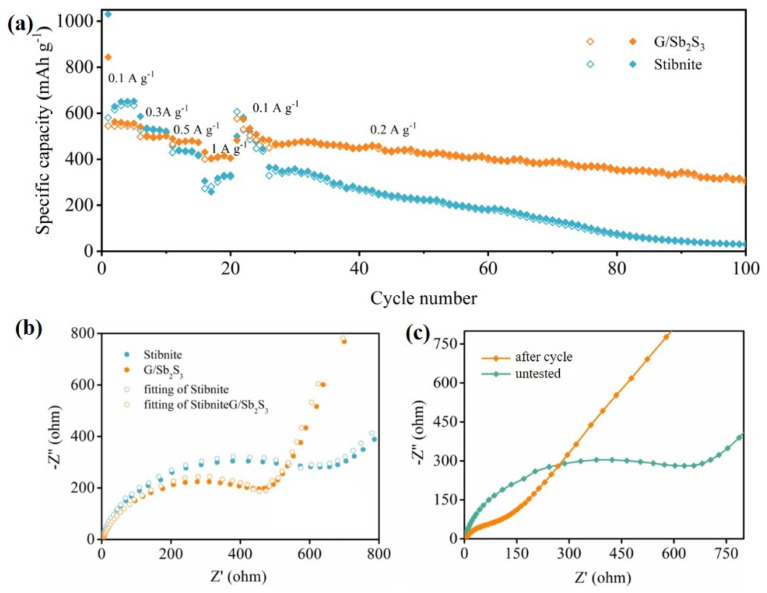
(a) Rate performance of the Sb_2_S_3_ electrode. (b) Nyquist plots of G/Sb_2_S_3_ and stibnite at the initial state. (c) Nyquist plots of G/Sb_2_S_3_ at different states. Reproduced in part from Mingxiang Deng *et al.*, *RSC Adv.*, 2019, **9**, 15210–15216.^1^

The corresponding text “Finally, the content of Sb_2_S_3_in the G/Sb_2_S_3_ composite was determined to be 66.68 wt%.” in the original article should be corrected as “Finally, the content of Sb_2_S_3_ in the G/Sb_2_S_3_ composite was determined to be 74.68 wt%.”

The corresponding text “The G/Sb_2_S_3_ composites delivered the average reversible capacities of 642.3, 507.6, 392.1 and 264.9 mA h g^−1^” in the original article should be corrected as “The G/Sb_2_S_3_ composites delivered the average reversible capacities of 544.9, 497.6, 474.5 and 407.0 mA h g^−1^”.

The corresponding text “the average reversible capacity of the G/Sb_2_S_3_ composites could recover to 595 mA h g^−1^ after 20 cycles” in the original article should be corrected as “the average reversible capacity of the G/Sb_2_S_3_ composites could recover to 576.7 mA h g^−1^ after 20 cycles”.

The corresponding text “After 75 cycles, the reversible capacity was 422 mA h g^−1^ with the capacity retention of 82.9%.” in the original article should be corrected as “After 75 cycles, the reversible capacity was 365.0 mA h g^−1^ with the capacity retention of 67%.”

The authors wish to draw the readers’ attention to their closely related *RSC Advances*^1^ paper, published at nearly the same time, which should have been cited in this *RSC Advances* paper.

Fig. 1–5 were re-used in part from ref. 1 without being correctly attributed. The corrected captions are shown below:

Fig. 1 (a) XRD patterns of G/Sb_2_S_3_ and stibnite. (b) TGA curves of G/Sb_2_S_3_ and stibnite. (c) Raman spectra of G/Sb_2_S_3_ and stibnite. (d) Raman spectra of Sb–S. Reproduced in part from Mingxiang Deng *et al.*, *RSC Adv*., 2019, **9**, 15210–15216.^1^

Fig. 2 (a) XPS survey of G/Sb_2_S_3_ and stibnite. The corresponding high-resolution spectra of Sb (b), S (c) and C (d). Reproduced in part from Mingxiang Deng *et al.*, *RSC Adv.*, 2019, **9**, 15210–15216.^1^

Fig. 3 (a and b) SEM image of stibnite. (c–f) SEM image of the G/Sb_2_S_3_ composites. (g and h) TEM image of the G/Sb_2_S_3_ composites. (i) HR-TEM image of the G/Sb_2_S_3_ composites. Reproduced in part from Mingxiang Deng *et al.*, *RSC Adv.*, 2019, **9**, 15210–15216.^1^

Fig. 4 Cyclic voltammograms of initial three cycles of (a) G/Sb_2_S_3_ composites, (b) stibnite. (c) Galvanostatic discharge/charge curves of G/Sb_2_S_3_ composites. (d) Cycling performance and coulombic efficiencies of G/Sb_2_S_3_ and stibnite. Reproduced in part from Mingxiang Deng *et al.*, *RSC Adv.*, 2019, **9**, 15210–15216.^1^

Fig. 5 (a) Rate performance of the Sb_2_S_3_ electrode. (b) Nyquist plots of G/Sb_2_S_3_ and stibnite at the initial state. (c) Nyquist plots of G/Sb_2_S_3_ at different states. Reproduced in part from Mingxiang Deng *et al.*, *RSC Adv.*, 2019, **9**, 15210–15216.^1^

The Royal Society of Chemistry apologises for these errors and any consequent inconvenience to authors and readers.

## Supplementary Material
